# Identification of the *DOG1* Gene Family in Cultivated Peanut and Negative Regulation of *Ralstonia solanacearum* Infection by *AhDOG1-L4*

**DOI:** 10.3390/biology15181662

**Published:** 2026-09-19

**Authors:** Yongli Zhang, Meiling Song, Yunxin Chen, Dongyuan Tian, Chong Zhang, Weijian Zhuang

**Affiliations:** 1 Center for Legume Plant Genetics and System Biology, College of Agriculture, Fujian Agriculture and Forestry University, Fuzhou 350002, China; zhang9745@outlook.com (Y.Z.); 18765747093@163.com (M.S.); alemon2233@163.com (Y.C.); tdy1558724079@163.com (D.T.); 2 Key Laboratory of Genetics, Breeding and Multiple Utilization of Crops, Ministry of Education, Fujian Agriculture and Forestry University, Fuzhou 350002, China; 3 Key Laboratory of Biological Breeding for Fujian and Taiwan Crops, Ministry of Agriculture and Rural Affairs, Fujian Agriculture and Forestry University, Fuzhou 350002, China; 4 Institute of Oil Crops Research, College of Agriculture, Fujian Agriculture and Forestry University, Fuzhou 350002, China

**Keywords:** peanut, bacterial wilt, phylogenetic relationship, plant defense

## Abstract

Bacterial wilt, caused by *Ralstonia solanacearum*, is a devastating soilborne disease often referred to as plant cancer. It threatens hundreds of species of plants and causes losses in yield and a deterioration in the quality of infected plants. Delay of Germination 1 (DOG1) plays vital roles in plant growth, development, and abiotic and biotic stress responses. However, the functions of *DOG1* in biotic stress remain unexplored in cultivated peanut (*Arachis hypogaea* L.). In this study, we systematically identified and characterized the *AhDOG1* gene family in peanut using bioinformatics approaches and used transgenic experiments to functionally investigate *AhDOG1-L4* in response to infection with *Ralstonia solanacearum*. A total of 37 *AhDOG1* genes were identified in the peanut genome and classified into six subfamilies based on their phylogenic relationship. Their patterns of expression were studied and showed that *AhDOG1-L4* is highly expressed in roots, root tips, rhizomes, nodules, stems, stem tips, cytoledons, leaves, florescences, and pegs. Importantly, among these tissues, the root tips exhibit the highest expression level of *AhDOG1-L4*. The heterologous overexpression of *AhDOG1-L4* significantly reduced the tolerance of transgenic *Arabidopsis thaliana* to *Ralstonia solanacearum* infection. Moreover, *AhDOG1-L4* may participate in plant defense against pathogens by modulating the expression of genes associated with defense signaling networks. Functional characterization revealed that heterologous overexpression of *AhDOG1-L4* in *Arabidopsis* significantly increased susceptibility to *Ralstonia solanacearum* by suppressing the expression of defense-related genes, demonstrating that *AhDOG1-L4* functions as a plant negative regulator of bacterial wilt resistance. To the best of our knowledge, this is the first study into the roles of *AhDOG1* genes in the responses to stress in peanut plants.

## Simple Summary

Bacterial wilt, caused by the pathogen *Ralstonia solanacearum*, is a serious soilborne disease that threatens peanut production worldwide, leading to significant yield losses. Identifying plant genes that influence disease resistance is essential for developing more resilient peanut varieties. In this study, we identified 37 members of the *DOG1* gene family in the peanut genome. Among them, we focused on a gene called *AhDOG1-L4*, which is highly active in roots—the main entry point for the pathogen. When we introduced this gene into the model plant *Arabidopsis thaliana*, the transgenic plants became more vulnerable to bacterial wilt and showed reduced activity of several defense-related genes. Our findings suggest that *AhDOG1-L4* acts as a negative regulator of disease resistance. This discovery provides new insights into how plants interact with pathogens and may offer a potential target for breeding peanut varieties with improved resistance to bacterial wilt.

## 1. Introduction

Cultivated peanut (*Arachis hypogaea* L., AABB, 2n = 4x = 40) is an allotetraploid derived from a hybridization between *Arachis duranensis* (2n = 2x = 20, AA genome) and *Arachis ipaensis* (2n = 2x = 20, BB genome) [1,2,3,4]. The value of peanut as a globally important oilseed crop had led to intensive research on improving its yield and quality to advance the peanut industry [5,6]. However, biotic stresses severely compromise the productivity of peanut plants. Bacterial wilt, caused by *Ralstonia solanacearum*, is a soilborne disease that affects over 450 species of plants worldwide, including economically vital crops, such as tobacco (*Nicotiana tabacum*), peanut, soybean (*Glycine max*), rapeseed (*Brassica napus*), tomato (*Solanum lycopersicum*), and pepper (*Capsicum annuum*), and causes yield losses of 10–50% or even complete crop failure [7,8]. *Ralstonia solanacearum* primarily infects plants through their roots. It produces abundant extracellular polysaccharides and toxins in the xylem vessels, which subsequently spread to the stems and throughout the entire plant, blocking water transport and ultimately leading to wilting and death [9,10,11]. No fully safe and effective control strategy has been established to date. Therefore, the current strategy to control this disease entails mining disease resistance genes to breed resistant cultivars.

Delay of Germination 1 (DOG1) encodes a heme-binding protein, and heme, an essential iron-porphyrin compound, acts as a ubiquitous cofactor in plants [12]. The *DOG1* gene family can be classified into five groups in *Arabidopsis thaliana*, including *DOG1* and *DOG1-Like 1–4* (*DOGL1–4*) based on their conserved domains [12]. *DOGL1*, *DOGL2*, *DOGL3*, and *DOGL4* share 54.3%, 43.1%, 39.3%, and 23.4% sequence similarity with *DOG1*, respectively [12]. Several studies have reported that *DOG1* is involved in plant defense. For example, in poplar (*Populus davidiana* × *P. alba var. Pyramidlis*, cv ‘Shanxin’), *PdPapDOL3,* a member of the *DOG1* family, plays a significant role in response to soilborne diseases [13]. Notably, *PdPapDOL3* is differentially expressed in the shoot tips, leaves, and roots 48 h after inoculation with various soilborne pathogens; similarly, the exogenous application of salicylic acid (SA), jasmonic acid (JA), and abscisic acid (ABA) markedly induces its differential expression across tissues [13]. Moreover, DOG1 may function indirectly in biotic stress. A total of 42 *BnDOG1* family members have been identified in rapeseed, and transcriptome data revealed that the *BnDOG1* genes respond to manganese stress [14]. Manganese contributes to lignin biosynthesis, which reinforces the strength and rigidity of plant cell walls and, in turn, suppresses plant disease [15,16]. These findings collectively imply that the *DOG1* family members operate within plant defense signaling networks. Although *DOG1* family members have been primarily characterized in *Arabidopsis* and cereal crops as central regulators of seed dormancy and abiotic stress adaptation via ABA signaling networks, emerging evidence suggests that seed-associated signaling hubs extensively cross-talk with innate immunity to balance growth, dormancy, and survival under pathogen attack [17]. Peanut production is severely constrained by soilborne vascular pathogens such as *Ralstonia solanacearum*. Investigating whether and how peanut *DOG1* homologs participate in biotic stress adaptation is therefore crucial for uncovering immune regulatory networks and identifying novel targets to alleviate susceptibility in cultivated peanut.

This study used bioinformatics to systematically identify the *AhDOG1* gene family at the level of the peanut genome. We then analyzed their structural variations, evolutionary relationships, and patterns of expression in more detail. This study used genetic transformation to demonstrate that the heterologous expression of *AhDOG1-L4* in *Arabidopsis thaliana* significantly reduces the level of resistance to *Ralstonia solanacearum*. This work provides a foundation for the future exploration of the *DOG1* gene family in plant defense signaling networks.

## 2. Materials and Methods

### 2.1. Identification of the DOG1 Gene Family in the Peanut Genomes

Data on the protein sequences of cultivated peanut (*Arachis hypogaea* cv. ′Shitouqi′), *Arachis duranensis*, and *Arachis ipaensis* were retrieved from the Peanut Genome Resource database (PGR, https://pgr.itps.ncku.edu.tw/, accessed on 15 June 2026). The hidden Markov model (HMM) profile of the DOG1 domain (PF14144) was obtained from the Pfam database (http://pfam.xfam.org/, accessed on 15 June 2026). HMMER 3.0 program [18] was utilized to search the peanut protein database with a threshold of 1 × 10^−5^ to identify proteins in the DOG1 family. The presence of the domain was verified further using the NCBI Conserved Domain Database (https://www.ncbi.nlm.nih.gov/cdd/, accessed on 15 June 2026), InterProScan (http://www.ebi.ac.uk/iterpro/search/sequence-search, accessed on 15 June 2026), and the SMART online tool (http://smart.embl-heidelberg.de/, accessed on 15 June 2026). Candidate proteins lacking the intact signature domain or displaying incomplete open reading frames were systematically excluded.

The theoretical isoelectric point (pI) and molecular weight (MW) values of the candidate proteins were predicted using the ExPASy online tool (http://web.expasy.org/compute_pi/, accessed on 18 June 2026). The subcellular localization of the proteins was predicted using the CELLO v.2.5 online server (http://www.csbio.sjtu.edu.cn/bioinf/Cell-PLoc/, accessed on 18 June 2026) [19].

### 2.2. Phylogenetic and Gene Structure Analysis

The evolutionary relationships among the *DOG1* family members in peanuts were studied. DOG1 protein sequences from the cultivated peanut, *Arachis duranensis*, *Arachis ipaensis*, and *Arabidopsis thaliana* [12] were used to construct a phylogenetic tree. MAFFT was used to align the multiple sequences [20]. The phylogenetic tree was constructed using the neighbor-joining method with 1000 bootstrap replications by the MEGA7.0 software [21].

The gene structures and conserved motifs of the *AhDOG1* family were analyzed using the TBtools software (v2.485) [22]. The exon–intron organization was visualized based on the GFF3 annotation of the cultivated peanut genome using TBtools (v2.485). The MEME suite (https://meme-suite.org/meme/index.html, accessed on 15 June 2026) was used to identify conserved motifs [23]. The maximum number of motifs was set to 20, and the other parameters were kept as default. The gene structure graphics were generated using TBtools (v2.485) [22].

The chromosomal positions of the *AhDOG1* genes were visualized using TBtools (v2.485) [22] according to their genomic coordinates.

### 2.3. Prediction of the Cis-Acting Elements in the Promoter Region of DOG1s

Promoter sequences of the *AhDOG1* genes that were 2 kb long were extracted. Stress-responsive *cis*-acting elements were systematically identified using the PlantCARE online database (http://bioinformatics.psb.ugent.be/webtools/plantcare/html/, accessed on 18 June 2026) [24]. The elements identified were visualized with TBtools (v2.485) [22].

### 2.4. Transcriptome-Based Patterns of Expression of the AhDOG1 Genes in Different Peanut Tissues

Transcriptome data from 10 different tissues/developmental stages were obtained from the PGR database [2]. The detailed transcriptomic dataset and different tissues’ developmental stages are explained in our previous publication [2]. The transcriptomic data was obtained from the public repository of PGR under the project accession number FAFU005-FAFU023. The levels of gene expression were quantified as log_2_-transformed fragments per kilobase of transcripts per million mapped reads (FPKM) values and visualized using TBtools (v2.485) [22]. All the genes were clustered using the complete linkage method with Euclidean distance measurement by the default parameters of the TBtools software (v2.485).

### 2.5. Construction of the Overexpression Vector and Transformation of Arabidopsis thaliana

The coding sequence of *AhDOG1-L4* was cloned from ‘Shitouqi’ peanut by reverse transcription PCR (RT-PCR). The primers used to amplify the PCR are listed in Table S1. The PCR conditions were as follows: 98 °C for 10 s, 55 °C for 5 s, and 72 °C for 30 s, for 35 cycles. An overexpression vector driven by the CaMV 35S promoter was constructed through BP (Gateway™ BP Clonase™ Enzyme Mix) and LR (Gateway™ LR Clonase™ Enzyme Mix); (Thermo Fisher Scientific (Invitrogen™), Carlsbad, CA, USA) recombination reactions and designated pK7WG2.0-*AhDOG1-L4*. The p35S::*AhDOG1-L4* construct was introduced into *Agrobacterium tumefaciens* strain GV3101.

The floral dipping method was used to generate transgenic *Arabidopsis thaliana* plants [25]. To confirm transgenic plants, the initial transgenic T_0_ and T_1_ lines were screened by Kanmycin and further confirmed by RT-PCR. Five independent T_1_ lines were selfed to generate T_2_ individual lines. After antibiotic screening and RT-PCR verification, three independent single-insertion homozygous and highly expressed T_3_ lines (designated as OE-6, OE-9, and OE-15) were confirmed by genomic PCR using gene- and vector-specific primers and validated for *AhDOG1-L4* transcript abundance via RT-PCR. Three homozygous T_3_ transgenic lines were selected as the functional characterization.

### 2.6. Subcellular Localization **Analysis**

The *AhDOG1-L4* coding sequence without the stop codon was cloned into the yellow fluorescent protein (YFP) fusion vector pFGC-eYFP to generate p35S::*AhDOG1-L4*-YFP (Table S1). The empty p35S::eYFP vector was used as a control. Both constructs were individually transformed into *Agrobacterium tumefaciens* GV3101. The specific plasma membrane marker p35S::AtPIP2A::mCherry was co-agroinfitrated with p35S::AhDOG1-L4-YFP and empty p35S::eYFP vector [26]. Subcellular localization were assessed by transient expression in *Nicotiana benthamiana* leaf epidermal cells in *Ralstonia solanacearum* infection and mock condition. For two conditions, 10 µL *Ralstonia solanacearum* and ddH_2_O were inoculated into the different plant leaf veins 12 h after *Agrobacterium* infiltration. A laser scanning confocal microscope (Leica TCS SP8, Leica, Solms, Germany) was used to image the YFP and mCherry fluorescence 48 h after agroinfiltration at excitation wavelengths of 514 and 561 nm, respectively.

### 2.7. Inoculation with Ralstonia solanacearum and Disease Resistance Assay

The resistance to disease was evaluated by inoculating 4-week-old transgenic *Arabidopsis thaliana* plants with *Ralstonia solanacearum* strain GMI1000. The bacterial strain was streaked on 2,3,5-triphenyltetrazolium chloride (TTC) agar medium (10 g/L peptone, 1 g/L casein hydrolysate, 5 g/L glucose, and 12 g/L agar, pH 7.2); (Solarbio Life Sciences, Beijing, China) and incubated at 28 °C for 48 h. A single colony was picked and cultured overnight in BG liquid medium (10 g/L peptone, 1 g/L yeast extract, 1 g/L casein hydrolysate, 5 g/L glucose); (Solarbio Life Sciences, Beijing, China) at 28 °C with shaking at 200 rpm. The bacterial culture was grown to an OD_600_ of approximately 0.8 in BG liquid medium. The bacterial cells were collected by centrifugation at 4000 rpm for 15 min, resuspended in distilled water, and adjusted to a final OD_600_ of 0.8. The roots were inoculated by creating standardized “#”-shaped mechanical wounds in the root zone with a sterile scalpel and then drenching each plant with 5 mL of the bacterial suspension. The inoculated plants were maintained in a highly humid growth chamber at 28 °C. The disease severity was recorded systematically and scored according to the following four-grade scale: grade 1, leaf curling; grade 2, petiole lodging; grade 3, leaf yellowing and desiccation; grade 4, death of the whole plant. The disease index (DI) was calculated using the following formula:

$$\mathrm{Disease}\;\mathrm{index}=\frac{\sum_{0}^{5}x_{i}y_{i}}{x_{\max}\sum y_{i}}\times 100\%$$

where *x_i_*: disease grade value; *x_max_*: the highest disease grade value; *y_i_*: the number of diseased plants corresponding to the disease rating. At 72 h post-inoculation, 1 g of leaf tissue was placed in a mortar and thoroughly ground with 5 mL of sterile distilled water to obtain a homogenate. The supernatant was collected and subsequently subjected to ten-fold serial dilutions. An aliquot (100 µL) of each dilution was spread onto BG + PB solid medium and incubated for 3 days. The resulting colonies of *Ralstonia solanacearum* were then enumerated.

### 2.8. RNA Extraction and qRT-PCR Analysis

The total RNA was extracted using the TRIzol reagent; (Sangon Biotech Co., Ltd., Shanghai, China) and reverse-transcribed into cDNA using an Advantage RT-for-PCR Kit (RR047A, Takara, Dalian, China), according to the manufacturer’s instructions. Quantitative real-time reverse transcription PCR (qRT-PCR) was performed for the *AtPR1*, *AtNPR1*, *AtMPK5*, *AtPDF2.1,* and *AtFMO1* genes related to defense. The *Actin* gene was used as an internal control to normalize the levels of expression. The relative gene expression was calculated using the 2^−ΔΔCt^ method [27]. All the qRT-PCR assays included three biological replicates for each sample. The gene-specific primers used for qRT-PCR are listed in Table S1.

The qRT-PCR was conducted on a Bio-Rad (Hercules, CA, USA) instrument with the following program: pre-denaturation at 95 °C for 5 min, followed by 40 cycles of 95 °C for 15 s and 60 °C for 30 s. A melting curve was generated from 65 °C to 95 °C after amplification.

### 2.9. Statistical Analysis

All experiments were conducted with at least three independent biological replicates. Pairwise comparisons between two genotypes were performed using an unpaired, two-tailed Student’s t-test. Statistical computations were performed in GraphPad Prism (v8.0.1). Error bars in the figures represent the SD calculated from three biological replicates. * *p* < 0.05. ** *p* < 0.01.

## 3. Results

### 3.1. The DOG1 Gene Family in Cultivated Peanut

A total of 37 *DOG1* genes were identified in the cultivated peanut genome, along with 19 and 18 *DOG1* genes in the *Arachis duranensis* and *Arachis ipaensis* genomes, respectively. Following the nomenclature of the *Arabidopsis thaliana* DOG1 family, the peanut *DOG1* genes were designated *AhDOG1-Like-1* (*AhDOG1-L1*)–*AhDOG1-Like-37* (*AhDOG1-L37*), *AdDOG1-Like-1* (*AdDOG1-L1*)–*AdDOG1-Like-19* (*AdDOG1-L19*), and *AiDOG1-Like-1* (*AiDOG1-L1*)–*AiDOG1-Like-18* (*AiDOG1-L18*) (Table S2).

The CDS lengths of the 37 *AhDOG1* genes varied. The longest were *AhDOG1-L1* and *AhDOG1-L18* (1515 bp), while the shortest was *AhDOG1-L10* (546 bp). The predicted pI values ranged from 5.1 (*AhDOG1-L4*) to 8.79 (*AhDOG1-L36*), and the MW values ranged from 20,640.41 Da (*AhDOG1-L10*) to 56,729.92 Da (*AhDOG1-L1*) (Table S3).

### 3.2. Phylogenetic Analysis of the DOG1 Proteins

**Figure 1:**
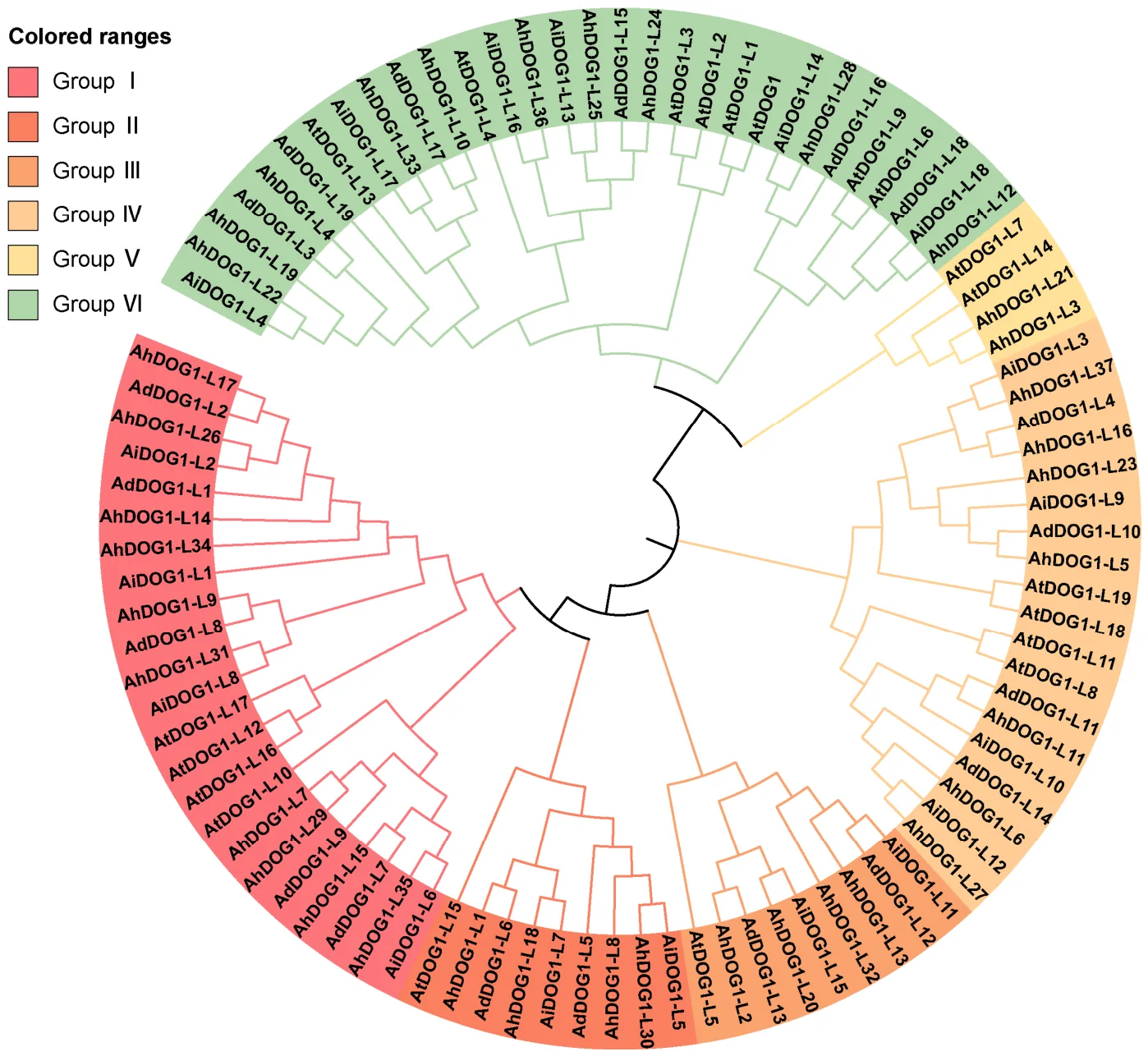
Phylogenetic relationships of the *DOG1* gene family from *Arachis hypogaea*, *Arachis duranensis*, *Arachis ipaensis,* and *Arabidopsis thaliana*. The phylogenetic tree was constructed using MEGA7 based on the neighbor-joining method with 1000 bootstrap replicates. There are six subfamilies in the *DOG1s* family. Different colored arcs mean different subfamilies.

**Figure 2:**
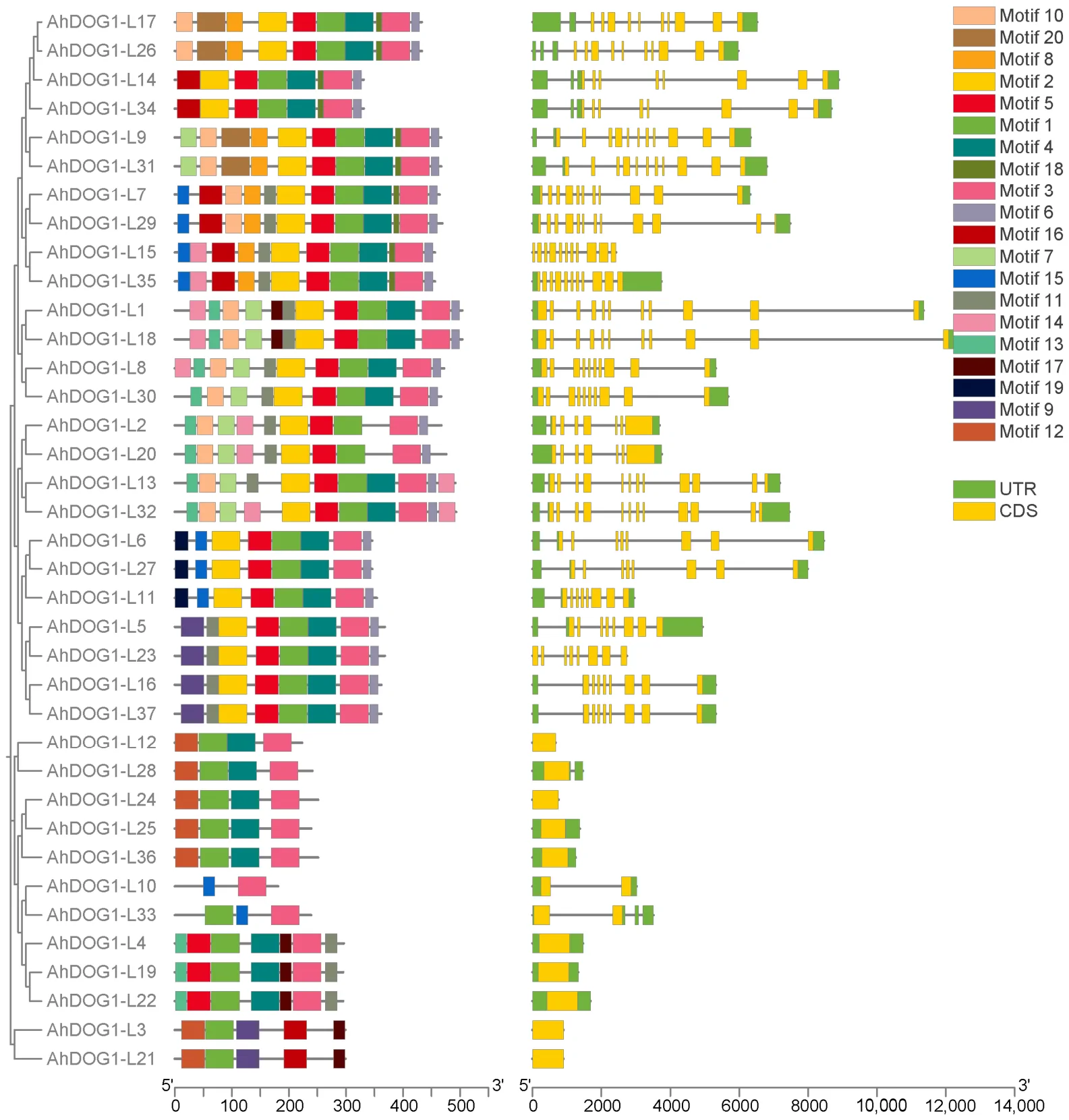
The motif and gene structure of *DOG1* genes in cultivated peanut. (**Left panel**) phylogenetic tree of *AhDOG1s* as shown in Figure 1. Middle panel, 20 conserved motifs distribution using online MEME software (https://meme-suite.org/meme/index.html, accessed on 15 June 2026). Boxes with different colors represent the diverse conserved motifs. (**Right panel**) exon–intron structure. Yellow boxes, exons; gray lines, introns; green boxes, untranslated regions.

The evolutionary relationships of the peanut *DOG1* gene family were elucidated in more detail by constructing phylogenetic trees based on the sequence information of 94 homologous genes from cultivated peanut, *Arachis duranensis* (Ad), *Arachis ipaensis* (Ai), and *Arabidopsis thaliana* (At), including 37 AhDOG1, 19 AdDOG1, 18 AiDOG1, and 20 AtDOG1. Phylogenetic analysis categorized the peanut DOG1 family into six well-defined subfamilies (Subfamilies I–VI), supported by the classification rule in *Arabidopsis* (Figure 1). This topological grouping is further corroborated by conserved exon–intron architecture and domain composition analysis (Figure 2), which revealed that members within the same evolutionary clade share highly congruent motif arrangements. Compared with the number of DOG1 in three species of *Arachis*, the number in cultivated peanut is equal to that of two diploid peanuts, suggesting that DOG1 is conserved in the three species of *Arachis*.

### 3.3. Chromosomal Distribution, Conserved Motif, and Gene Structure of the AhDOG1 Genes

**Figure 3:**
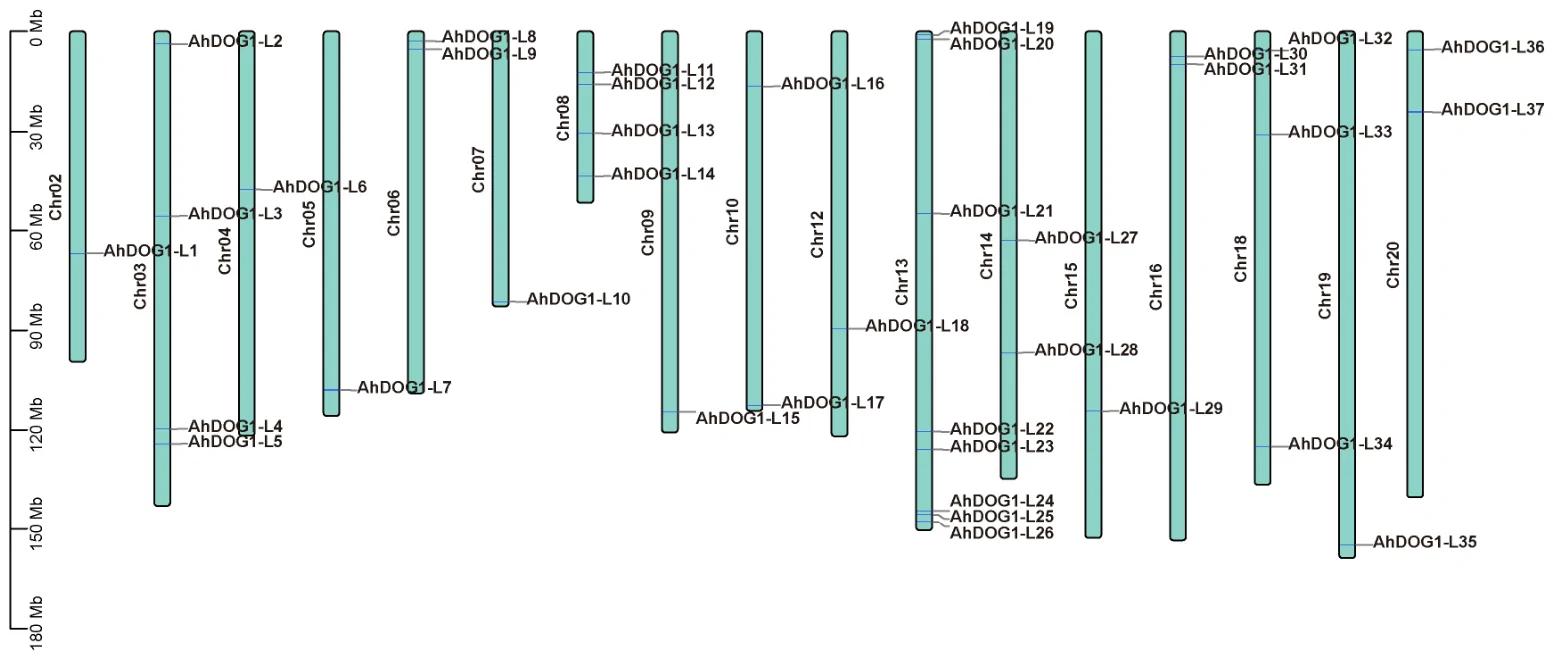
The chromosomal distribution of *AhDOG1s* in cultivated peanut. The scale at the left side of figure is shown in Mb. The location of *AhDOG1s* is indicated on one side of each chromosome.

The variation in structure among the *AhDOG1* family members was systematically analyzed by examining their chromosomal distribution, conserved motifs, and gene structures. The 37 *AhDOG1* genes were unevenly distributed across 17 chromosomes. No *AhDOG1* genes were detected on chromosomes 1, 11, or 17. Chromosome 13 harbored the largest number of *AhDOG1* (eight genes), whereas chromosomes 2, 4, 5, 7, 9, 12, 15, and 19 each carried a single gene (Figure 3). The *AhDOG1* genes were preferentially located at the ends of chromosomes.

The MEME tool was used to identify 20 conserved motifs in the AhDOG1 proteins (Table S4). The motif composition was highly consistent within the same subgroup of the phylogenetic tree (Figure 2). For example, subgroup I contained motifs 2, 5, 1, 4, 18, 3, and 6, and subgroup II contained motifs 13, 10, 7, 11, 2, 5, 1, 4, and 3. Moreover, motifs 20, 8, and 18 were specific to subgroup I, and motif 19 was exclusive to subgroup IV. Notably, motif 1 was present in all the AhDOG1 sequences and motif 3 in most of them; thus, motif 1 is universally conserved in all AhDOG1 members, while its functional significance requires further experimental verification.

The exon–intron structure revealed that genes with similar patterns of exon–introns tended to cluster together (Figure 2). The number of introns in the 37 *AhDOG1* genes ranged from 0 to 12. Subgroups I, II, III, IV, V, and VI contained 9–12, 9–10, 6–12, 7–8, 0, and 0–3 introns, respectively (Figure 2). The genes with the largest intron counts were *AhDOG1-L26*, *AhDOG1-L13*, and *AhDOG1-L32* (12 introns), whereas *AhDOG1-L12*, *AhDOG1-L24*, *AhDOG1-L25*, *AhDOG1-L36*, *AhDOG1-L4*, *AhDOG1-L19*, *AhDOG1-L22*, *AhDOG1-L3*, and *AhDOG1-L21* had fewer introns.

### 3.4. Cis-Acting Elements in the AhDOG1 Promoters

**Figure 4:**
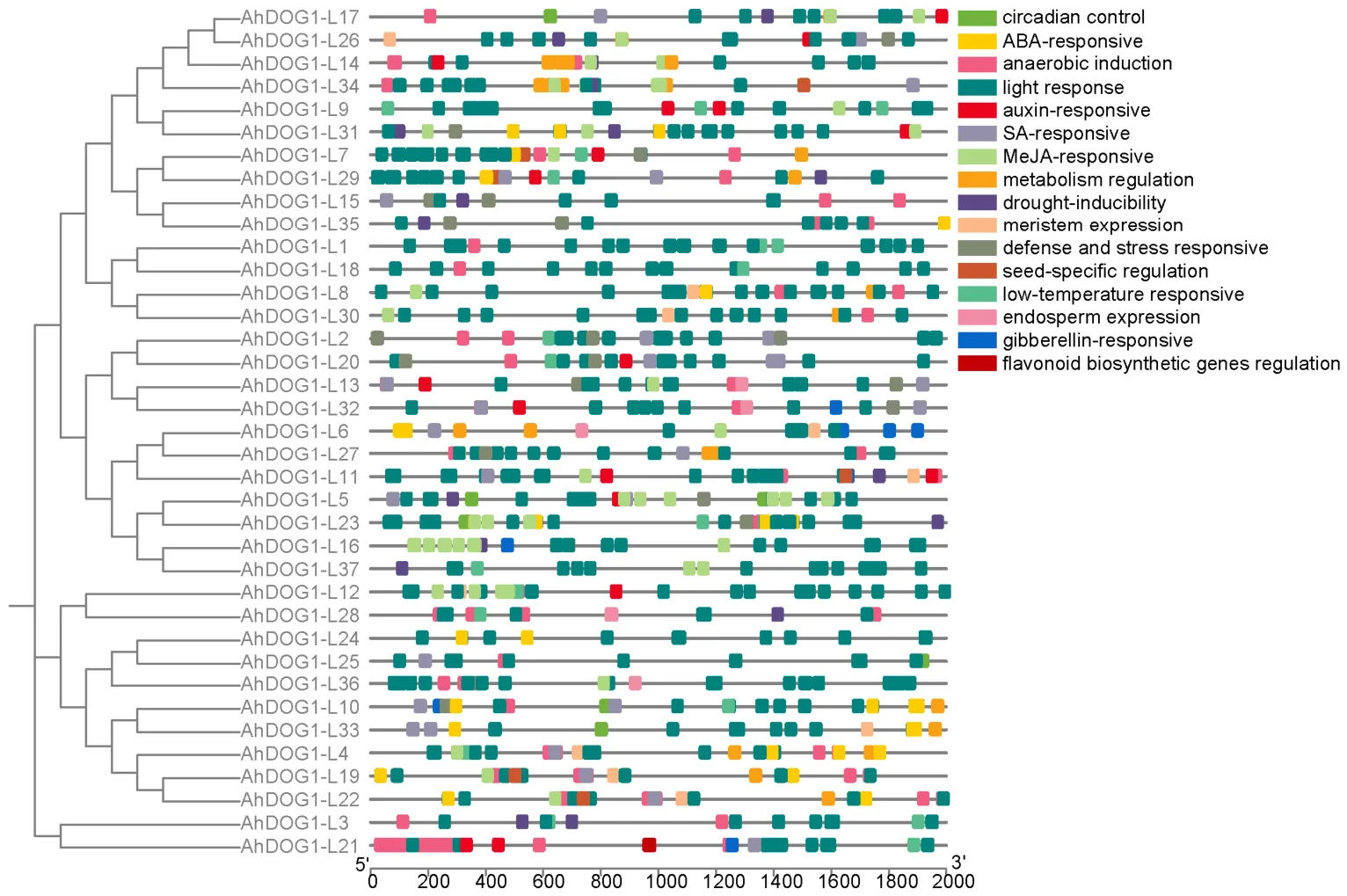
Putative *cis*-acting elements in the promoter regions of the *AhDOG1*. The *cis*-acting elements were predicted in the PlantCARE (http://bioinformatics.psb.ugent.be/webtools/plantcare/html/, accessed on 18 June 2026). Diverse-colored boxes indicate different regulatory responsive elements.

The PlantCARE database was used to identify the *cis*-acting elements in the 2 kb promoter regions of the *AhDOG1* genes. A total of 16 types of stress-related *cis*-acting elements were detected, including those responsive to light, low temperature, drought, defense and stress, methyl jasmonate (MeJA), ABA, auxin, gibberellin, SA, and anaerobic induction (Figure 4). Light-responsive elements were present in all the *AhDOG1* promoters. Anaerobic induction elements were found in 30 genes, ABA-responsive elements in 24 genes, MeJA-responsive elements in 22 genes, and SA-responsive elements in 20 genes. Defense/stress-, drought-, auxin-, and low-temperature-responsive elements were each found in 15 genes. A small number of members also harbored elements related to regulation specific to the meristems and seeds. These results indicate that the *AhDOG1* family members are probably involved in the development and diverse stress responses of peanuts.

### 3.5. Patterns of Expression of the AhDOG1 Genes in Different Peanut Tissues

**Figure 5:**
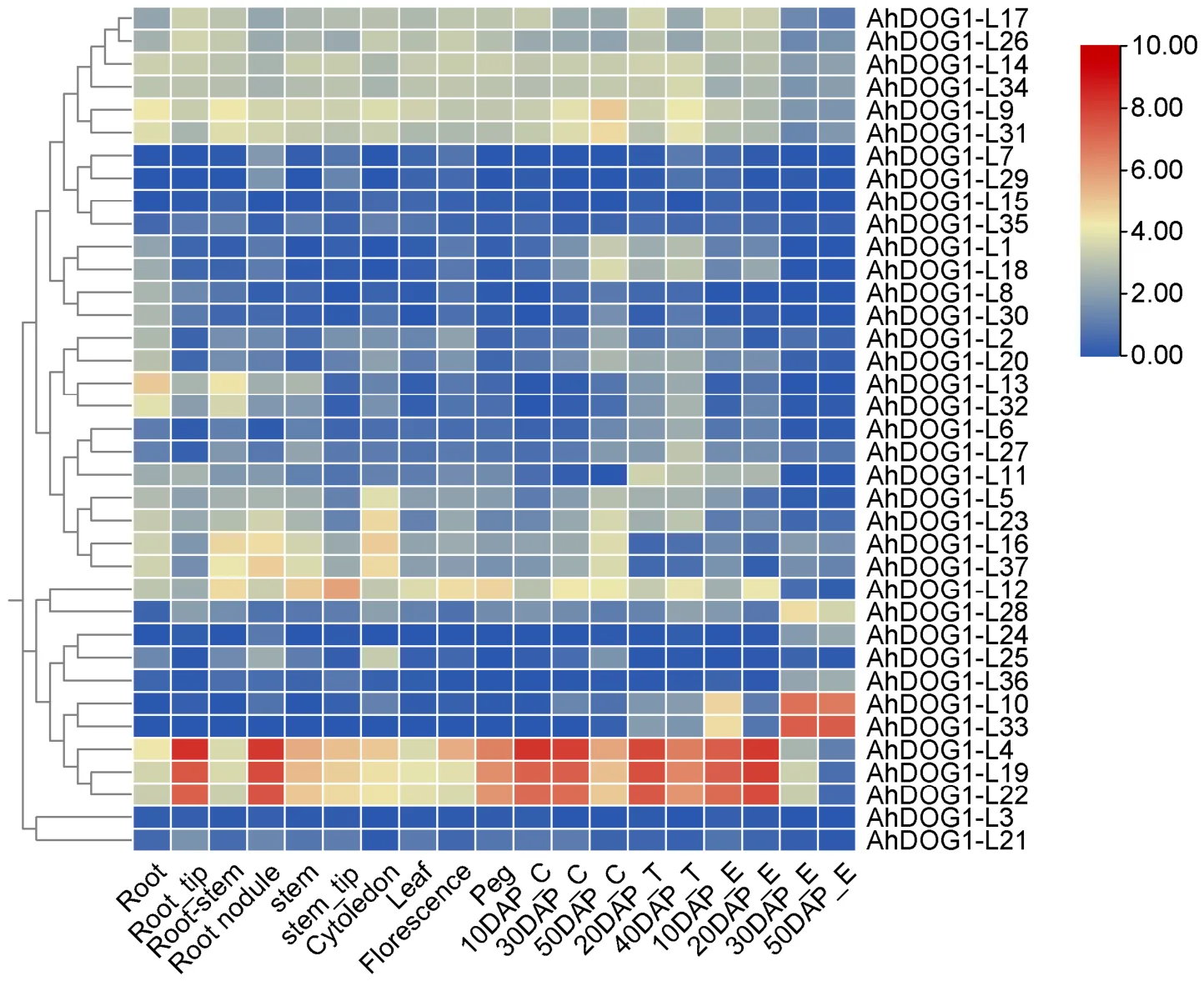
Heatmap shows the pattern of expression of *AhDOG1* genes in different tissues and developmental stages of the seeds of cultivated peanut. The patterns of expression of the 37 *AhDOG1* genes are displayed by hierarchical clustering based on RNA-seq data. The upper color scale indicates log_2_-based fragments per kilobase of transcript per million mapped reads (FPKM). The genes were clustered using the complete linkage method with Euclidean distance measurement. Root, root tips, stem, stem tips, root stem, leaf, inflorescence, peg, cotyledon, and root nodules indicated different tissues in the development stages. 10DAP_E, 20DAP_E, 30DAP_E, and 50DAP_E indicate embryos from plants of pod development and mature stages, including four stages of 10, 20, 30, and 50 days after pegging; 10DAP_C, 30DAP_C, and 50DAP_C indicate pericarps from plants of pod development stage and mature stages, including 10, 30, and 50 days after pegging; 20DAP_T and 40DAP_T indicate testa from plants of pod development and mature stages, including two stages of 20 and 40 days after pegging. The FPKM value is shown in Table S5.

Publicly available transcriptome data was used to explore the potential functions of the *AhDOG1* genes by analyzing their patterns of expression across various tissues and developmental stages (Figure 5 and Table S5). All 37 *AhDOG1* genes were expressed in the tissues examined, and 21 were detected in all the tissues tested. These findings suggest that these genes have broad functional roles in the development of peanut. Low (FPKM ≤ 1), moderate (1 < FPKM < 10), and high (FPKM ≥ 10) levels of expression were identified based on a previous study [28]. Three genes, including *AhDOG1-L4*, *AhDOG1-L19*, and *AhDOG1-L22*, were highly expressed throughout the development of embryos, seed coats, and pericarps, which indicates that these genes are probably involved in the development of seeds. Notably, *AhDOG1-L4* was highly expressed overall across the tissues tested, and the highest levels were identified in the roots, root tips, rhizomes, nodules, stems, and stem tips. We hypothesized that *AhDOG1-L4* might function when the pathogen invades the roots and colonizes the xylem and, therefore, employed a transgenic approach to validate its function.

### 3.6. Subcellular Localization of AhDOG1-L4

**Figure 6:**
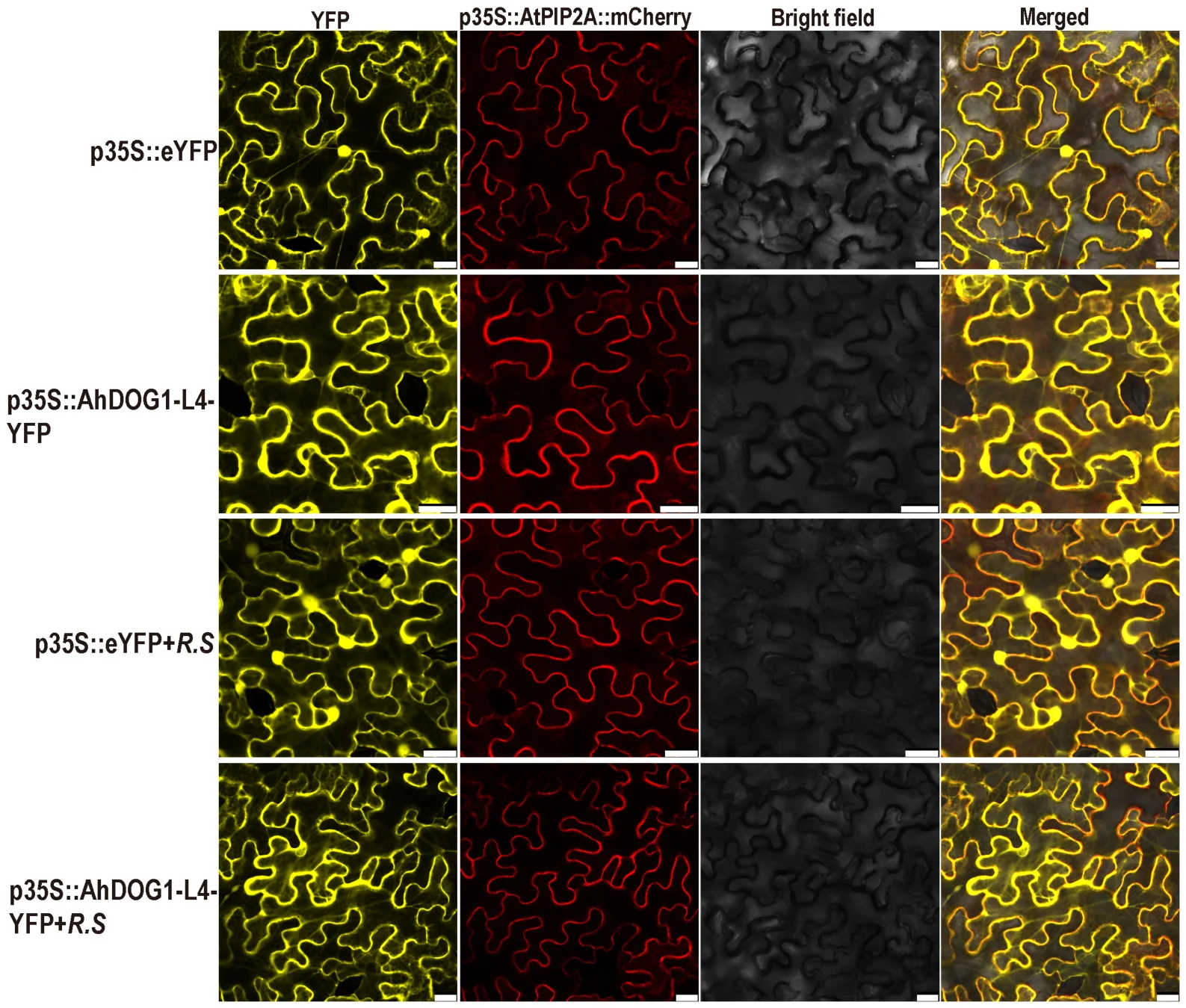
Subcellular localization of AhDOG1-L4 in the epidermal cells of *Nicotiana benthamiana* leaves using confocal fluorescence microscope. YFP, control; AtPIP2A::mCherry, specific plasma membrane marker; 35S::YFP + *R.S* and 35S::AhDOG1-L4::YFP + *R.S*, agroinfiltrated leaf by the fluorescence proteins were inoculated into the leaf veins 12 h after *Agrobacterium* infiltration separately. The fusion constructs were transiently expressed in *Nicotiana benthamiana* leaves, followed by inoculation with *Ralstonia solanacearu* after 12 h. Fluorescence (left), bright field (middle), and merged images were obtained at 48 h using Leica confocal microscopy after agroinfiltration. Bar: 25 µm. YFP, Yellow fluorescent protein.

An *AhDOG1-L4*::YFP fusion construct driven by the 35S promoter and the YFP empty vector control were transiently expressed in the leaves of *N. benthamiana*. The YFP was distributed throughout the entire cell, whereas the *AhDOG1-L4*::YFP was detected in the cytoplasm, plasma membrane, and nucleus (Figure 6). These results demonstrate that *AhDOG1-L4* localizes to the cytoplasm, plasma membrane, and nucleus, where it may have important functions. Furthermore, following transient expression in tobacco leaves and subsequent infiltration with *Ralstonia solanacearum*, the subcellular localization pattern of *AhDOG1-L4*::YFP remained unaltered.

### 3.7. Heterologous Overexpression of AhDOG1-L4 in Arabidopsis thaliana Reduced Its Tolerance to R. solanacearum

**Figure 7:**
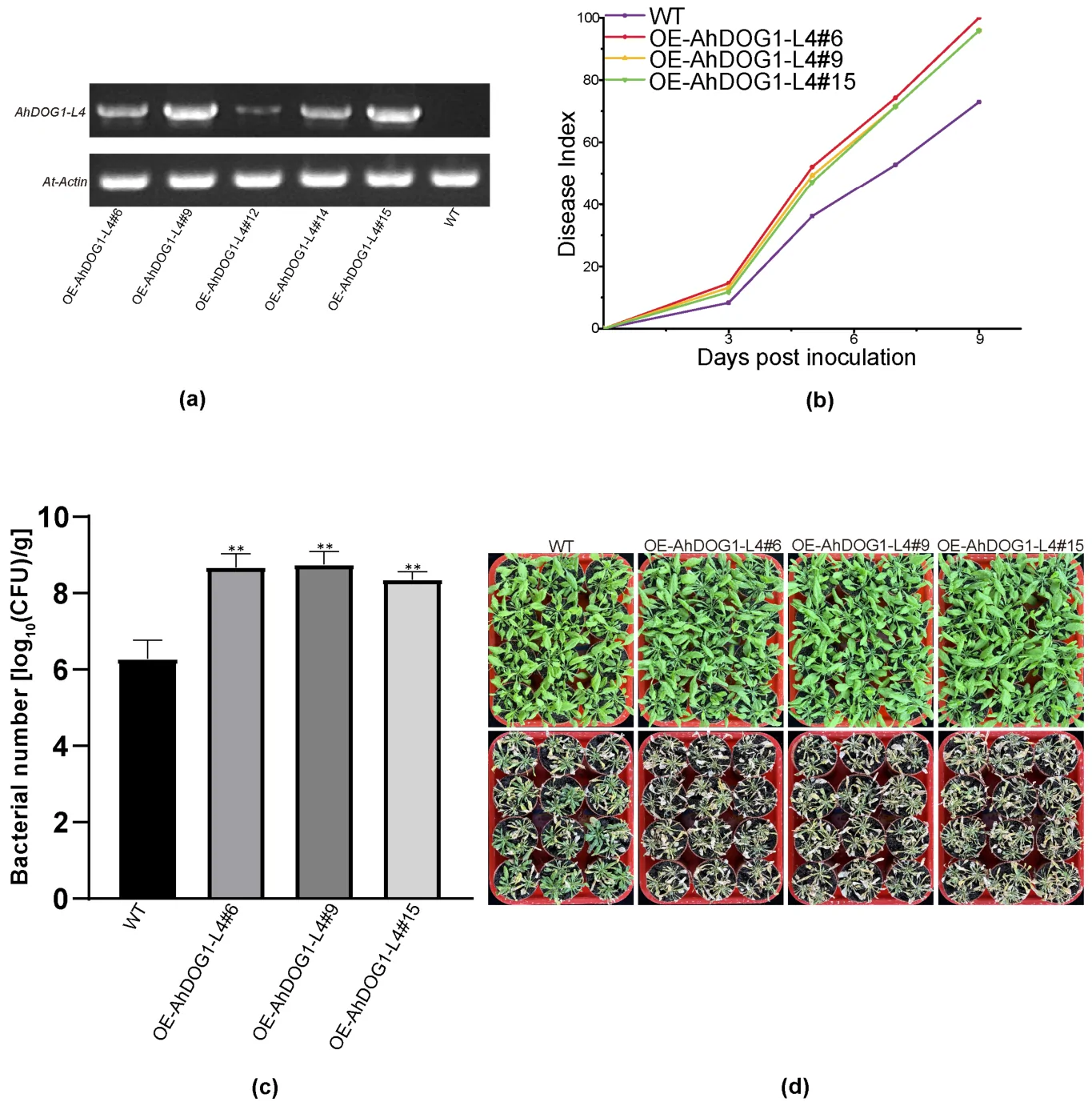
Overexpression of *AhDOG1-L4* reduced resistance to *Ralstonia solanacearum* in transgenic *Arabidopsis thaliana*. (**a**) RT-PCR analysis of *AhDOG1-L4* genes in transgenic *Arabidopsis thaliana*. (**b**) Disease indices of different OE lines and the WT after inoculation with *Ralstonia solanacearum*. (**c**) Bacterial titers in *Arabidopsis thaliana* were quantified at 3 dpi with *Ralstonia solanacearum*. (**d**) The root-drenching method was used to inoculate 4-week-old WT and transgenic *Arabidopsis* plants with 5 mL of bacterial suspension per plant. The photograph was obtained 9 days post-inoculation (dpi). OE, Overexpression; WT, wild type. Statistical significance was evaluated using a *t*-test analysis. Error bars, SD calculated from three biological replicates. ** *p* < 0.01.

*AhDOG1-L4* was overexpressed in *Arabidopsis thaliana* to investigate the potential function of the *AhDOG1* gene family under *Ralstonia solanacearum* stress. Three independent transgenic lines (Figure 7a) and wild-type (WT) plants were inoculated with *R. solanacearum*, and the resistance to disease was assessed by the disease index. The WT and transgenic plants began to show disease symptoms after 3 days post-inoculation (dpi). The disease index of WT was 8.3%, whereas those of the three transgenic lines were 14.5%, 13.1%, and 11.8%. The incidence of disease in the WT reached 72.9%, while the transgenic lines displayed rates of 100%, 95.8%, and 95.8% after 9 dpi (Figure 7b). Comparison of the colony counts of *Ralstonia solanacearum* in wild-type and *AhDOG1-L4* transgenic *Arabidopsis* at 72 h post-inoculation revealed that the transgenic line harbored significantly higher bacterial titers than the wild type (Figure 7c). These results demonstrate that the overexpression of *AhDOG1-L4* enhanced the susceptibility of *Arabidopsis thaliana* to *Ralstonia solanacearum* (Figure 7d).

### 3.8. Overexpression of AhDOG1-L4 Reduced the Levels of Expression of Defense-Related Genes in Arabidopsis thaliana Under Bacterial Infection

**Figure 8:**
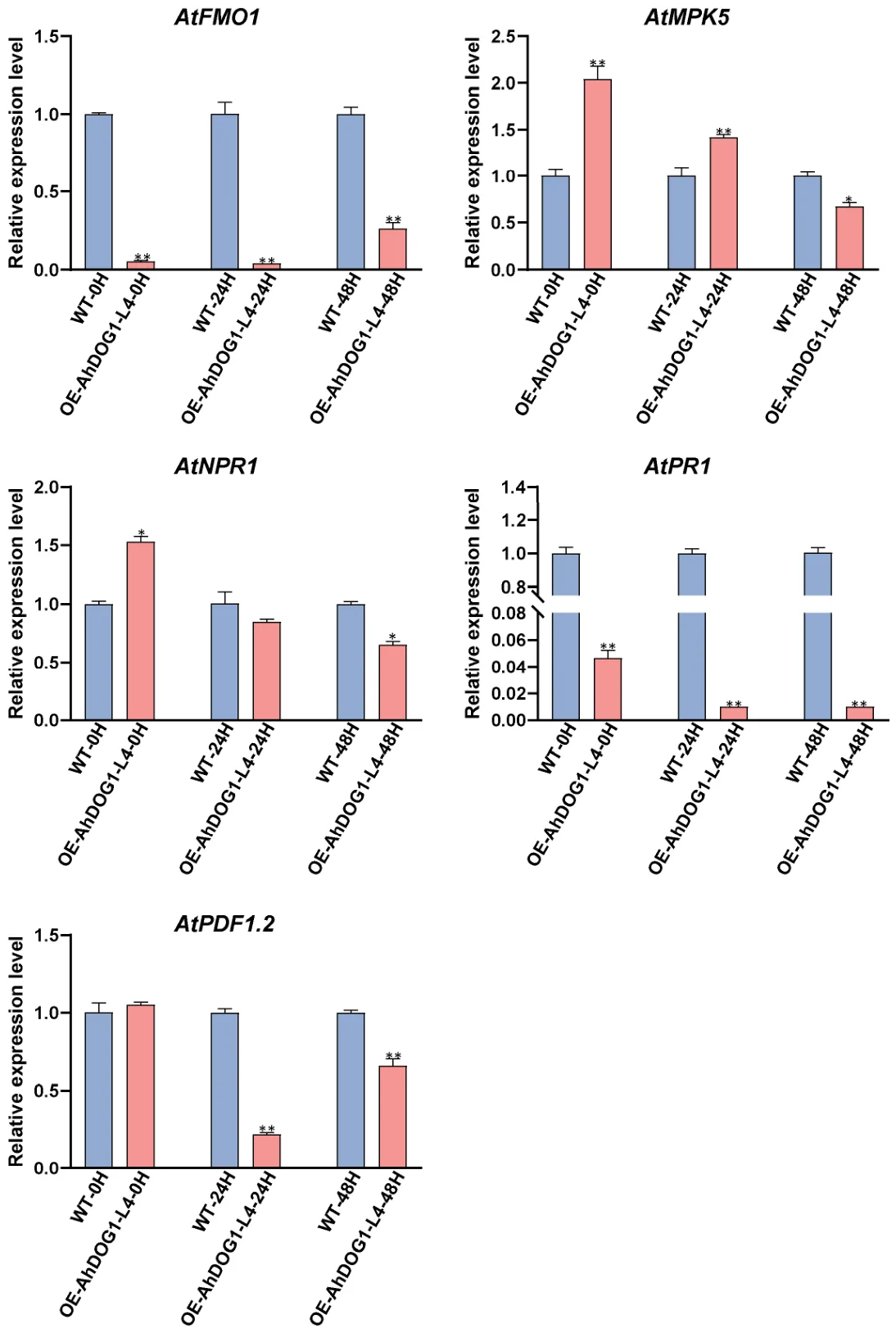
Transcript levels of the defense marker genes in transgenic or non-transgenic *Arabidopsis thaliana* after inoculation of *Ralstonia solanacearum* based on qRT-PCR. There were three independent repetitions of the biological experiments. Statistical significance was evaluated using a *t*-test analysis. Error bars, SD calculated from three biological replicates. * *p* < 0.05. ** *p* < 0.01. qRT-PCR, Real-time quantitative reverse transcription PCR.

The mechanism underlying the increased susceptibility mediated by *AhDOG1-L4* was examined in more detail using qRT-PCR to study the patterns of expression of key defense signaling marker genes at 0, 24, and 48 h post inoculation (hpi) with *Ralstonia solanacearum*. At 24 h post-inoculation (hpi), the SA pathway marker gene *PR1* was significantly downregulated approximately 100-fold in the transgenic lines compared to the WT, whereas there was no significant difference in the expression of *AtNPR1*. At 48 h, *AtPR1* and *AtNPR1* were downregulated approximately 100-fold and 1.5-fold, respectively. The jasmonic acid/ethylene (JA/ET) pathway core marker gene *AtPDF1.2* was downregulated 4.5-fold and 1.5-fold at 24 hpi and 48 hpi, respectively. The expression of *AtMPK5*, a key gene in the mitogen-activated protein kinase (MAPK) cascade, was initially upregulated and then downregulated compared with the WT. Moreover, the systemic acquired resistance (SAR) pathway core gene *AtFMO1* was downregulated 25-fold and 3.8-fold at 24 h, and 48 h, respectively (Figure 8). Collectively, upon *Ralstonia solanacearum* infection, the genes associated with defense, including *AtMPK5*, *AtNPR1, AtPR1*, *AtPDF1.2,* and *AtFMO1* were significantly downregulated at 48 hpi in the transgenic lines compared to the WT plants.

## 4. Discussion

As a globally important economic and oil crop, peanut plays an irreplaceable role in food security, nutritional improvement, and rural development. However, increasingly severe bacterial wilt caused by *Ralstonia solanacearum* poses a grave threat to its yield and quality. Thus, there is an urgent need to identify the key genes within defense signaling networks. *DOG1* genes have been identified in multiple plant species, including *Arabidopsis thaliana*, soybean, wheat (*Triticum aestivum* L.), pepper, rapeseed, and Moso bamboo (*Phyllostachys edulis*) [12,14,29,30,31,32]. In this study, we used an HMM-based approach to identify 37 *AhDOG1* genes in cultivated peanut. Notably, the total number of *DOG1* genes in cultivated peanut equals the sum of those in the two wild diploid progenitor species. Among the identified family members, 21 *AhDOG1* genes were constitutively expressed across all surveyed vegetative and reproductive tissues. Given the allotetraploid nature of cultivated peanut, several of these genes exist as homoeologous pairs originating from the A and B subgenomes. Their broad expression patterns, combined with conserved domain architectures, suggest substantial functional redundancy in basal cellular regulation. However, subtle divergences in regulatory promoter elements and differential responses to environmental stimuli suggest that, while baseline functions are preserved, certain duplicated pairs may undergo subfunctionalization in response to specialized developmental or stress cues. In this study, we found that *AhDOG1-L4* is highly expressed in various tissues, but it negatively regulated infection by *Ralstonia solanacearum* by damaging the immune system in transgenic *Arabidopsis thaliana* plants.

To date, research on the *DOG1* family has focused almost exclusively on seed dormancy, the regulation of germination, and abiotic stress responses. The *DOG1* family is ubiquitous in angiosperms and exists in multiple copies even in model plants, such as *Arabidopsis thaliana* [12,33]. Its divergence from its ancestral role in seed dormancy [12] has spawned a repertoire of functions associated with development and abiotic stress tolerance, including flowering [34], drought and heat tolerance [35,36,37,38], manganese stress [32], and salt stress [39]. However, little was known about its role in resistance to plant disease. To the best of our knowledge, this study provides the first evidence that *AhDOG1-L4* significantly enhances susceptibility to *Ralstonia solanacearum* when heterologously expressed in *Arabidopsis thaliana*, thus filling a conspicuous gap in the biological functions of this gene family under biotic stress. This discovery compels a fundamental reassessment of the role that the *DOG1* genes may play in defense signaling networks. Therefore, the observation of this phenomenon may represent a newly evolved function in biotic stress responses acquired during the evolutionary history of the *DOG1* family.

The overexpression of *AhDOG1-L4* led to a marked increase in mortality when the plants were inoculated with *Ralstonia solanacearum*. We propose two mechanistic explanations. First, the TGA family of bZIP transcription factors (TFs), as core components of plant innate immunity and SAR [40,41], interacts with NPR1 through their DOG1 domains in the nucleus to form the NPR1-TGA transcriptional complex [42]. This interaction enhances the TGA factors to bind DNA and activates the pathogenesis-related genes that respond to SA [43], thus underscoring the essential role of the DOG1 domain in resistance to pathogens. Critically, the *DOG1* gene family and TGA TFs share this identical DOG1 domain. This structural convergence strongly suggests that the DOG1 family proteins may similarly participate in the recognition of pathogens or downstream defense regulation through this domain. We speculate that the observed disease susceptibility in *Arabidopsis thaliana* could arise from competitive interference. For example, the DOG1 domain might also interact with NPR1, thereby disrupting the formation of functional TGA-NPR1 complexes and attenuating the defense signaling mediated by SA. Secondly, the DOG1 proteins could directly bind a *Ralstonia* effector, which would accelerate invasion and colonization by the pathogen or be hijacked by the pathogen to suppress the host defense network. Although the *DOG1* family has not been previously implicated in plant disease resistance, the shared conserved DOG1 domain with the TGA factors logically positions the AhDOG1 members as potentially important participants in plant–pathogen interactions. They may act as negative regulators that increase susceptibility or, under certain circumstances, positively contribute to defense signaling. This hypothesis not only opens new perspectives to understand the biological functions of the *AhDOG1* family but also identifies potential new targets to improve crop disease resistance. All these hypotheses require further experimental validation.

When the plants perceive a pathogen, they rapidly assemble a complex regulatory network in which multiple defense signaling pathways are interconnected and function both independently and synergistically. Each pathway has characteristic marker genes whose transcript levels sensitively reflect its status of activation [44,45]. We utilized qRT-PCR to compare the expression of key marker genes in *AhDOG1-L4* transgenic and WT *Arabidopsis thaliana* after infection with *Ralstonia solanacearum*. The bacterial stress resulted in significant downregulation of the SA pathway core genes *AtPR1* and *AtNPR1*, thus indicating the suppression of SA signal transduction. This suppression could conceivably be one of the key strategies employed by the pathogen to breach the host’s basal immunity. *AtPDF1.2*, which encodes an antimicrobial peptide and serves as a quintessential molecular marker of the JA/ET pathway, was markedly repressed in transgenic plants. This repression indicates a failure to activate this immune branch response and produce antimicrobial peptides, thereby facilitating pathogen infection. *AtFMO1* is a central gene in the SAR pathway; it catalyzes the biosynthesis of *N*-hydroxypipecolic acid (NHP), which orchestrates the transduction of SA and JA signals, induces the expression of genes related to immunity, and amplifies systemic resistance [46,47]. The pronounced downregulation of *AtFMO1* in transgenic plants indicates a critical disruption in the SAR pathway that blocks the biosynthesis of NHP and prevents the establishment of SAR. *AtMPK5*, a member of the MAPK cascade, functions as a core hub that integrates biotic and abiotic stress and hormone signaling. Its upregulation at 24 hpi reflects an initial attempt by transgenic plants to mount a defense against the invading pathogen mediated by MAPK. However, this response failed to effectively restrict the proliferation and spread of the pathogen. Following infection with *Ralstonia solanacearum*, the expression of defense-related proteins was significantly suppressed in *AhDOG1-L4* transgenic *Arabidopsis*, ultimately resulting in a susceptible phenotype.

From an agronomic perspective, the identification of *AhDOG1-L4* as a susceptibility factor opens promising avenues for peanut breeding. Because negative regulators can be inactivated to confer broad-spectrum resistance, targeted editing of *AhDOG1-L4* or its promoter region via *CRISPR*/Cas9 could enhance resistance to bacterial wilt in elite peanut cultivars. Whether the negative regulatory role of *AhDOG1-L4* is restricted to *Ralstonia solanacearum* or confers broader susceptibility to fungal pathogens or abiotic stress remains to be determined. Systematic challenge assays involving diverse pathogens and osmotic stresses will be essential to establish the functional specificity of *AhDOG1-L4*.

## 5. Conclusions

In summary, this study identified the *DOG1* gene family in the peanut genome and used a bioinformatic analysis to reveal the variation in gene structure and evolutionary relationships. Expression profiling highlighted *AhDOG1-L4* as a potentially pivotal gene. Functional characterization demonstrated that AhDOG1-L4 localizes to the cytoplasm, plasma membrane, and nucleus and confers increased susceptibility to bacterial wilt. The analysis of expression further indicated that *AhDOG1-L4* potentially disrupts defense signaling by affecting the levels of expression of *AtPR1*, *AtNPR1*, *AtPDF1.2*, *AtFMO1*, and *AtMPK5*, thereby compromising the plant resistance to *Ralstonia solanacearum*. However, it should be noted that the functional validation presented in this study was performed in a heterologous *Arabidopsis thaliana* system. The endogenous role of *AhDOG1-L4* in peanut and the hypothesized molecular mechanisms, such as competitive NPR1 binding or interaction with effectors, merit further experimental study. Nonetheless, these findings provide novel insights and a robust foundation for the future exploration of disease resistance mediated by DOG1 and its application in the genetic improvement of peanuts.

## Data Availability

The original contributions presented in this study are included in the article. Further inquiries can be directed to the corresponding authors.

## Supplementary Materials

The following supporting information can be downloaded at: https://www.mdpi.com/article/10.3390/biology15181662/s1. Table S1: Primers used in this study; Table S2: The protein names and the sequences of DOG1s in *Arabdopsis thaliana*, *Arachis hypogaea*, *Arachis ipaensis,* and *Arachis duranensis*; Table S3: Features of the identified *AhDOG1s* genes in cultivated peanut; Table S4: Detailed sequences of 20 AhDOG1s conserved motifs; Table S5: Expression profile of *AhDOG1s* in different tissues and seed developmental stages of cultivated peanut.

## Abbreviations

The following abbreviations are used in this manuscript:

DOG1	Delay of Germination 1
DOGL1–4	DOG1-Like 1–4
SA	Salicylic acid
JA	Jasmonic acid
ABA	Abscisic acid
HMM	Hidden Markov model
MW	Molecular weight
FPKM	Fragments per kilobase of transcripts per million mapped reads
TTC	2,3,5-triphenyltetrazolium chloride
Ad	*Arachis duranensis*
Ai	*Arachis ipaensis*
At	*Arabidopsis thaliana*
MeJA	Methyl jasmonate
WT	Wild type
dpi	Days post-inoculation
MAPK	Mitogen-activated protein kinase
SAR	Systemic acquired resistance
NHP	*N*-hydroxypipecolic acid
